# Zukunft des deutschen Wirtschaftsmodells

**DOI:** 10.1007/s10273-020-2754-8

**Published:** 2020-10-24

**Authors:** Überblick

**Keywords:** A10, E00, H00

## Abstract

30 Jahre nach der Deutschen Wiedervereinigung — und unter dem Eindruck der akuten Corona-Krise — fand der siebte New Paradigm Workshop des Forum New Economy zur Zukunft des deutschen Modells vom 28. bis 30. September in Berlin statt. Renommierte deutsche und internationale Experten diskutierten die „Zukunft des deutschen Wirtschaftsmodells”. Wie gut ist Deutschland noch auf die kommenden Herausforderungen vorbereitet? In diesem Zeitgespräch sollen die auf dem Workshop präsentierten Studien vorgestellt werden, die mit Unterstützung des Forum New Economy erstellten wurden, unter anderem zur Entwicklung der Ungleichheit in Deutschland, einer neuen Industriepolitik, der Relevanz fi skalpolitischer Regeln und den Tücken des deutschen Exportmodells. Ergänzt werden diese Beiträge durch eine Übersetzung des Konferenzbeitrags von Thomas Piketty.

## Abstract (en)

Thirty years after German reunification — and under distress due to the acute coronavirus crisis — the seventh New Paradigm Workshop of the Forum New Economy took place in Berlin on 28–30 September. Renowned German and international experts discussed the “Future of the German Economic Model”. How well is Germany prepared for the coming challenges? This forum features a selection of articles funded by the Forum New Economy and presented at its workshop, including studies on the development of inequality in Germany, new industrial policy, the relevance of fiscal rules and the pitfalls of the German export model. The forum also includes a translation of the keynote by Thomas Piketty.

